# Effect of adding various supplements on physicochemical properties and starch digestibility of cooked rice

**DOI:** 10.1038/s41598-024-75847-7

**Published:** 2024-10-19

**Authors:** Lin Wang, Yidi Cai, Peeraphat Prempree, Rili Hao, Dahai Jiang, Loraine Bainto-Ancheta, Yukiharu Ogawa

**Affiliations:** 1 Graduate School of Horticulture, Chiba University, 648, Matsudo, 271-8510 Chiba Japan; 2 College of Food Science and Engineering, Dalian Ocean University, No.52, Heishijiao St., Shahekou, Dalian, 116023 Liaoning People’s Republic of China; 3 Key Laboratory of Food Nutrition and Human Health in Universities of ShandongCollege of Food Science and Engineering, Shandong Agricultural University, Taian, 271018 People’s Republic of China; 4 Institute of Food Science and Technology, University of the Philippines Los Baños, 4031, College, Laguna, Philippines

**Keywords:** Cooked rice, Rapeseed oil, Chili pepper, Wasabi, In vitro starch digestibility, Fourier transform infrared (FTIR) spectroscopy, Physicochemical property, Nutrition, Carbohydrates

## Abstract

This study investigated the physicochemical modifications of cooked rice caused by adding various supplements (rapeseed oil, dried wasabi powder, and dried chili pepper powder). The physicochemical and digestive properties of treated cooked rice were analyzed using multiple techniques to determine the impact of supplements on the rice quality, including its starch digestibility. All samples with added supplements showed an increase in surface firmness (0.77–0.95 kg·m/s^2^ (N)) and a decrease in thickness (2.23–2.35 mm) and surface adhesiveness (1.43–7.22 J/m^3^). Compared to the control group, two absorption peaks at 2856 and 1748 cm^−1^ and new signals at 1683 and 1435 cm^−1^ appeared in the Fourier transform infrared (FTIR) spectroscopy. Analysis of FTIR results revealed that the interaction force was mainly through noncovalent interactions. Moreover, adding supplements increased the resistant starch (RS) levels in all samples. Scanning electron microscopy (SEM) suggested that oil-enriched phases, proteins, and polyphenols could cause large agglomeration and loose gel structure. These results suggested the formation of amylose-guest molecule complexes, which may influence starch functionality. Our work could provide insight into the starch–supplement interactions and the key factors affecting starch digestibility.

## Introduction

Rice is one of the most important agricultural products due to its high starch content and has been extensively studied as an energy source for humans^1^. Generally, starch digestibility varies due to differences in genetic type and processing strategies such as chemical and physical modification^2^. The starch digestion process involved the enzymatic depolymerization of starch into glucose monomers. Different guest molecules (e.g., amino acids, glycerol, lipids, non-starch polysaccharides, peptides, polyphenols, proteins, etc.) can be trapped within or between hollow helices of starch, forming amylose complexes with diverse nanometric dimensions, which leaded to variations in rate of starch digestion^2–7^.

Oil-starch complexes could form type 5 resistant starch (RS_5_), a new type of starch primarily formed through hydrophobic interaction^8^. Based on previous studies, a more order single helical structure could form between lipid molecules and the amylose cavity, resulting in starch digestion rate variations^3^. Previous work^9^ reported that cooked rice supplemented with rice bran oil showed the highest resistant starch content and the lowest in vitro digestion hydrolysis rate. The interactions between fatty acids present in the lipids and amylose were also confirmed by the FTIR spectral fingerprint spectrum within the 950 to 1200 cm^−1^ region^9^. However, the effects of lipids on the digestive properties of starch systems and the mechanism related to the presence of other food ingredients have not been thoroughly studied.

Proteins contain many hydrophilic groups, such as carboxyl, hydroxyl, amide, and thiol in alkyl side chains, enabling them to interact with starch molecules^10^. For example, Indica rice starch exhibits hydrophobic molecular interactions with soy protein/whey protein isolate and demonstrates hydrophobic, hydrogen bonding, and electrostatic interactions with casein. Thermal treatment further enhanced the formation of a three-dimensional gel network on a micron scale, which significantly influenced the microstructure of the complexes^11^. The physicochemical properties and digestion rate were also affected by polyphenols binding to starch through hydrogen bonds and hydrophobic interactions. These interactions may vary depending on the types and concentrations of both polyphenols and starch^12^. Previous studies demonstrated the resistant starch content increased when proanthocyanidins from high-tannin sorghum were complexed with potato starch due to the formation of type II intrahelical V-complexes^13^. Tea polyphenols have also been demonstrated to decrease the onset temperature (To), peak temperature (Tp), and conclusion temperature (Tc) of rice starch, whereas caffeic acid showed no impact on these parameters^14,15^.

Wasabi and red chili peppers are popular seasonings in cooking but serve as health-promoting substitutes due to their high levels of nutritional compounds and antioxidant properties^16,17^. They also contain rich nutritional components, including polyphenols, protein, vitamin C, dietary fiber, and flavonoids^16,18^. Their epidemiological and health advantages have thus been studied. However, their mechanism of influence on the susceptibility of starch to enzymatic hydrolysis by digestion in their presence remains insufficiently studied.

This study aims to explore the interactions between starch and various supplements, including rapeseed oil (RRS), dried chili pepper powder (CRS), and dried wasabi powder (WRS), by examining their short-range structures, morphological characteristics, physicochemical properties, and starch hydrolysis. The multi-scale structures and molecular interaction forces of the obtained complexes were characterized using combined analytical techniques. Differences in the resistant starch content and digestion kinetic behaviors related to the mechanism of in vitro digestion were also examined.

## Results and discussion

### Textural properties

The firmness, adhesiveness, and thickness of cooked rice grains mixed with different supplements are shown in Table 1. The cooked rice without any supplement had the lowest surface firmness (SF), whereas the SF of rice grains increased when additives were present (*p* < 0.05). Under high temperature and moisture conditions during cooking, rice grains can develop surface cracks as a result of water absorption and expansion of starch granules^19^. These cracks facilitate the entry of water molecules into the starch, promoting further starch gelatinization^20^. In addition, a decreased gelatinization enthalpy was reported as polyphenols, proteins, and other nutritional ingredients added, The interaction through hydrogen bonds, hydrophobic interactions, or other molecular forces may result in the attachment of these molecules to the grain surface, forming a gel network that can alter the firmness, adhesiveness, and digestibility of the starch^21^. Previous study incorporated oil into tapioca starch, and after heat treatment, the gel network was compared with native starch^22^. Their results showed that the physical entanglements of the starch molecular chains were strengthened, and the firmness of the three-dimensional gel network was promoted, inhibiting the swelling of starch. This phenomenon was also supported by the lower thickness of RRS (2.23 ± 0.10 mm) compared to CT (2.23 ± 0.10 mm). The effect of caffeic acid on maize starch was evaluated by previous study^23^ and found that caffeic acid improved the solubility and swelling power of the sample compared with native maize starch. In contrast to CRS (0.80 ± 0.14 N) and WRS (0.77 ± 0.03 N), the SF of RRS significantly increased to 0.95 ± 0.08 N, which can be explained mainly by water evaporation during the equilibrium period. Water migration from inside to outside was further promoted by the low moisture content of the dried chili pepper powder and dried wasabi, resulting in quick moisture loss on the surface of the rice grains, which might contribute to a higher SF. The effect of moisture transfer on SF was demonstrated by previous report^24^. They found out that rice grains stored at 90 °C exhibited a harder surface compared to grains stored at 30 °C. This was attributed to continuous and quicker water transfer from the core to drying surface layers, resulting in higher SF, which was consistent with our findings. Simultaneously, the evaporation of water, swollen space architecture of starch granules, and capillary pressure difference provided more space for oil absorption^25^. The oil absorption phenomenon was reported by previous study^26^, which quantified the oil distribution between tigernut starch and tigernut oil by confocal laser scanning microscope (CLSM) image. The oil adsorption capacity of the matrix rose from 1.10 g/100 g to 1.83/100 g as the temperature increased from 80 °C to 140 °C, which is related to the heat-induced pressure difference between starch and oil. Previous report also indicated adding oil to tapioca starch not only formed a well-linked gel network structure but also partially restored the disrupted starch gel network through the oil-starch combination, which could be explained by the higher SF of RRS in our current study^22^. However, the change in overall firmness (OF) showed the opposite trend to the surface firmness. Harder cores of rice grains were observed in CRS (18.14 ± 1.53 N) and WRS (17.88 ± 0.91 N), which might be related to continuous water migration from the rice grain to the powder. In contrast, RRS (15.02 ± 1.11 N) showed a significantly softer core compared to CT (16.43 ± 2.08 N), which could be associated with the triglycerides in oil, which could form a lipid-enriched phase as a physical barrier, retaining the moisture within the grains, thus resulting in a soft core^27,28^. For the surface adhesiveness (SA), the slight reduction in CRS and WRS was attributed to the physical barrier effect of the chili pepper powder and wasabi powder. The RRS showed the lowest SA (1.43 ± 0.25 J/m^3^), which could be explained by the formation of the lipid-enriched phase. Unlike the incomplete coverage of powder coating, oil fully coated the surface of rice grains and fully blocked the viscosity of starch due to its fluidity^27^. Previous study^29^ reported similar findings where the surface adhesiveness (SA) of rice grains decreased significantly after pre-frying with oil. However, the authors did not find any correlation between adhesiveness and damaged starch, indicating that the reduction in SA was solely attributed to the presence of oil in the surface layer rather than alterations in the starch itself. Overall adhesiveness (OA) did not show significant differences among the samples, with the lowest OA observed in RRS (0.23 ± 0.29 kJ/m^3^). The changes in OA and SA were considered due to the physical barrier of the supplements.

**Table 1:** Firmness, adhesiveness, and thickness of intact cooked rice grains with various supplements.

Sample	Firmness		Adhesiveness		Thickness (mm)
	Surface (*N*)	Overall (*N*)	Surface (J/m^3^)	Overall (kJ/m^3^)	
CT	0.74 ± 0.11 a	16.43 ± 2.08 ab	8.43 ± 0.47 d	0.39 ± 0.71 c	3.33 ± 0.28 a
RRS	0.95 ± 0.08 b	15.02 ± 1.11 a	1.43 ± 0.25 a	0.23 ± 0.29 a	2.23 ± 0.10 a
CRS	0.80 ± 0.14 ab	18.14 ± 1.53 b	6.55 ± 0.38 b	0.33 ± 0.48 bc	2.35 ± 0.07 a
WRS	0.77 ± 0.03 ab	17.88 ± 0.91 ab	7.22 ± 0.11 c	0.29 ± 0.20 ab	2.30 ± 0.14 a

*CT* cooked rice, *RRS* cooked rice added with rapeseed oil, *CRS* cooked rice added with dried chili pepper powder, *WRS* cooked rice added with dried wasabi powder.

Mean ± standard deviation. The sample number (n) is 4–5. Different lower-case letters in the same column indicate significant differences (*p* < 0.05).

### Moisture content

The moisture content (MC%) is summarized in Table 2. The cooked rice grain with dried wasabi and red chili pepper powder showed lower moisture content (48.22 ± 1.18% w.b. and 48.49 ± 1.00% w.b.) than the control (54.90 ± 0.85% w.b.). The decrease in MC% might be attributed to the lower MC% of dried wasabi and red chili pepper powder. In contrast, rice grains added with oil showed the least reduction of MC% (52.63 ± 1.06% w.b.), which might be due to the surface lipid layers affecting the evaporation of water^22^.

**Table 2:** Effect of adding various supplements on the moisture, crude proteins (CP), and resistant starch (RS) contents of cooked rice grains.

Sample	CP (% d.b.)	Moisture content (% w.b.)	RS (%)
CT	6.83 ± 0.02 b	54.90 ± 0.85 c	0.69 ± 0.04 a
RRS	6.40 ± 0.03 a	52.63 ± 1.06 b	0.79 ± 0.04 b
CRS	7.58 ± 0.03 c	48.49 ± 1.00 a	0.83 ± 0.03 b
WRS	8.10 ± 0.02 d	48.22 ± 1.18 a	0.81 ± 0.06 b

Mean ± standard deviation. The sample number (n) was as follows: CP (*n* = 4–6); moisture (*n* = 4–6); RS (*n* = 3–4).

*CT* cooked rice, *RRS* cooked rice added with rapeseed oil, *CRS* cooked rice added with dried chili pepper powder, *WRS* cooked rice added with dried wasabi powder, *CP* crude protein, *RS* resistant starch.

### Crude protein (CP) content

The CP of cooked rice grains with different supplements is expressed in Table 2. The CP content of CT was 6.83% d.b., which is similar to the variety of Kirara 397 (*Oryza sativa* L.) (7.93% d.b.) reported in a previous study^30^. The difference between these two samples might be related to the variety, cultivation environment, and cooking method. The CP content of other samples ranged from 6.40% d.b. to 8.10% d.b., which was significantly influenced by the different additions of supplements. RRS showed the lowest CP content whereas WRS showed the highest CP content. Previous study^30^ also found similar results, reporting that rice cooked with rapeseed oil had a lower CP content of 7.70% d.b. compared to the control group (7.93% d.b.). This decrease was attributed to the naturally low CP content in rapeseed oil and the breakdown of proteins into nitrogen-containing aroma compounds^31^. Meanwhile, the CP content of CRS and WRS were significantly increased by the addition of chili pepper powder and wasabi powder. This could be explained by proteins being prominent biomolecules present in seed material^19^. Changes in protein content from chili powder are also contributed by various factors such as drying treatments, raw materials, and processing methods^18^.

### Starch hydrolysis during in vitro gastro-small intestinal digestion

**Fig. 1:**
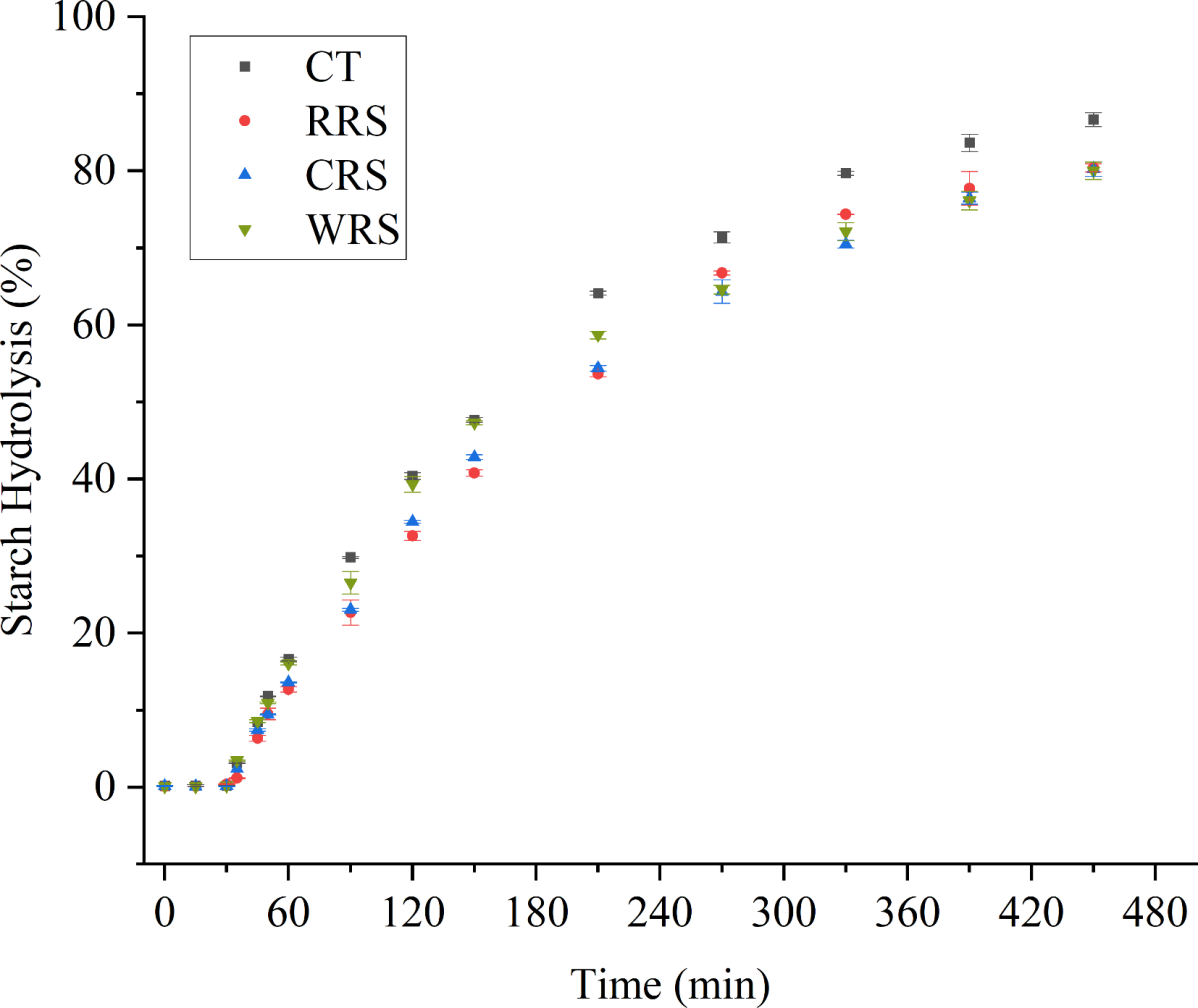
Changes in starch hydrolysis (%) of intact cooked rice grains with various supplements. Error bars represent standard deviation (*n* = 3–6). *CT* cooked rice, *RRS* cooked rice added with rapeseed oil, *CRS* cooked rice added with dried chili pepper powder, *WRS* cooked rice with added dried wasabi powder.

The hydrolysis of cooked rice starch with different supplements was measured as depicted in Fig. 1. Starch digestibility can be influenced by many factors, including starch sources, molecular structure, crystallinity, and modification^32^. During simulated gastric digestion, there was almost no observable starch hydrolysis, which aligns with findings from earlier studies^33^. This lack of hydrolysis is likely due to the absence of starch-hydrolyzing enzymes in the simulated gastric fluid. Similar results were observed^34^. On the other hand, it was suggested that the physical properties of starch and supplements could be altered by the digestive environment of the stomach^35^. All rice samples exhibited reduced digestion patterns compared to CT during the simulated small intestine digestion process, possibly due to the interactions between starch and various guest molecules. Previous study^36^ reported that the accessibility of enzymes to starch might be reduced by matrix compounds such as protein, lipids, and phenolics, thus, affecting starch digestibility. Other study^37^ elucidated the formation of amylose-lipid complexes when cooking rice with palm oil, noting an increase observed at a peak of 20° 2θ, indicative of V-type complexes. Meanwhile, the increased melting enthalpy and resistance starch content also contributed to the reduced in vitro starch digestibility. Additionally, previous work^38^ reported that natural starch completely disintegrated during cooking, whereas starch mixed with oil maintained a granular shape. This suggested that the starch structure could be reinforced by cross-linked bonds formed with oil. Prior studies reported that the oil-enriched phase on rice grains could cause agglomeration and adhesion of starch granules, facilitated by hydrophobic lipids. This phenomenon stabilized the starch gel network on the surface, restricting further leaching of amylose and accessibility of digestive enzymes, thereby reducing starch digestibility^3^. Similar results were observed in our experiment, in which a reduction of starch digestibility was shown in RRS (80.42%) compared with CT (86.66%). It is worth noting that RRS showed a lower degree of starch hydrolysis than CRS and WRS at the small intestine stage until 180 min, which might be due to the intact form of starch granules and presence of surface lipid layers. In addition, the higher starch hydrolysis degree is probably due to the emulsification of oil in digestive juices, which reduces the physical barrier effect to enzymes^35^. Other work also reported enhanced digestive breakdown of lipids, suggesting that bile salts, acting as natural surfactants (Na^+^, K^+^, and PO_4_^+^), facilitated easier exposure of starch encapsulated within lipids^35^. In contrast, the higher enzyme resistance of CRS and WRS in the later stages (I 180 min-I 420 min) may be due to the release of phenolic compounds. Previous report quantified the impact of phenolics on rice starch digestion by incorporating phenolics extracted from pigmented brown rice and red rice into white rice^9^. They found a reduction in starch hydrolysis ranging from 9.2 to 10.7%, highlighting phenolics as significant matrix compounds that contribute to lowering the glycemic response. The relationship between polyphenols and lower rates of digestion was also reported, noting a significant reduction in glucose release level following the addition of red grape^12^. Furthermore, they found a positive correlation (*R* = 0.9854) between the concentration of red grape polyphenols and the decreased digestibility of white rice, underscoring the potential role of phenolics in modulating glycemic response. Similar roles of polyphenols in modulating the in vitro digestibility of starch have also been reported from various berry sources^39^.

### Resistant starch (RS) content

To further reveal the glycemic potential of different samples, the resistant starch (RS) content was calculated (Table 2). After adding supplement, the resistant starch increased in all samples. Similar results were observed in white rice starch^37^, in which RS content increased from 18.90 to 23.90% after coking with palm oil. CRS and WRS showed a higher RS content than RRS, which might be attributed to the easier accessibility of polyphenols while the interaction between starch and fatty acids was obstructed by triglyceride macromolecules and oil-rich layers^22^. Previous research reported that the RS content in rice increased from 13.29 to 36.29% as the binding contents of proanthocyanidins (extracted from Chinese berry leaves) increased, indicating that the combination with proanthocyanidins was beneficial to the conversion of starch to RS, meanwhile, digestive enzyme activity was also inhibited by the released proanthocyanidins during digestion^39^. The difference in RS content might be attributed to the different sources and release speeds of nutrient substances inside the supplements and the interaction between starch and starch granule morphology^40,41^.

### Kinetics of starch digestibility

The kinetic parameters assessing differences during small intestinal digestion, including hydrolysis rates calculated using a non-linear fitting method with a first-order equation model, are summarized in Table 3. All kinetic parameters were significantly affected by the addition of different supplements. RRS showed higher $\:{\text{C}}_{{\infty\:}}$ of 94.92%, while its k was the lowest among all samples, which might be related to lower digestibility in the prophase of the small intestine due to the physical barrier of oil and reinforced structure by cross-linking^22^. The CRS and WRS had the lower $\:{\text{C}}_{{\infty\:}}$ of 89.64 and 83.49%, respectively, while the CRS showed a higher k (0.68) than WRS (0.53), suggesting that starch digestibility could be affected by the sources and release rates of phenols and other nutrients^41^. All first-order equation models exhibited high R^2^, suggesting that starch hydrolysis was well described. eGI was determined to simulate the level of glucose release. All samples showed significantly lower eGI compared with CT, suggesting that the addition of lipids and phenols in carbohydrate-rich foods might be a practical means to reduce the glycemic response of starchy food consumed globally.

**Table 3:** Kinetic parameters of starch hydrolysis of intact cooked rice grain cooked with various supplements.

Sample	$\:{\text{C}}_{{\infty\:}}$ (%)	k×10^−2^ (min^−1^)	*R* ^2^	HI	eGI
CT	93.35 ± 1.55 c	0.63 ± 0.02 c	0.99	68.80 ± 0.57 c	77.48 ± 0.31 c
RRS	94.92 ± 2.28 c	0.48 ± 0.02 a	0.99	61.05 ± 0.85 a	73.23 ± 0.46 a
CRS	89.64 ± 1.54 b	0.53 ± 0.02 b	0.99	60.76 ± 0.57 a	73.07 ± 0.31 a
WRS	83.49 ± 1.69 a	0.68 ± 0.03 d	0.99	63.65 ± 0.63 b	74.65 ± 0.34 b

Mean ± standard deviation. The sample number (n): $\:{\text{C}}_{{\infty\:}}$ (*n* = 3-6); k (*n* = 3-6); HI (*n* = 3–6); eGI (*n* = 3-6).

*CT* cooked rice, *RRS* cooked rice added with rapeseed oil, *CRS* cooked rice added with dried chili pepper powder, *WRS* cooked rice added with dried wasabi powder, $\:{\text{C}}_{{\infty\:}}$ equilibrium concentration of hydrolyzed starch, *R*^2^ determination coefficient, *k* kinetic constant, *HI* hydrolysis index, *eGI* estimated glycemic index.

Different letters within the same column indicate significant differences (*p* < 0.05).

### Short-range ordered structure

**Fig. 2:**
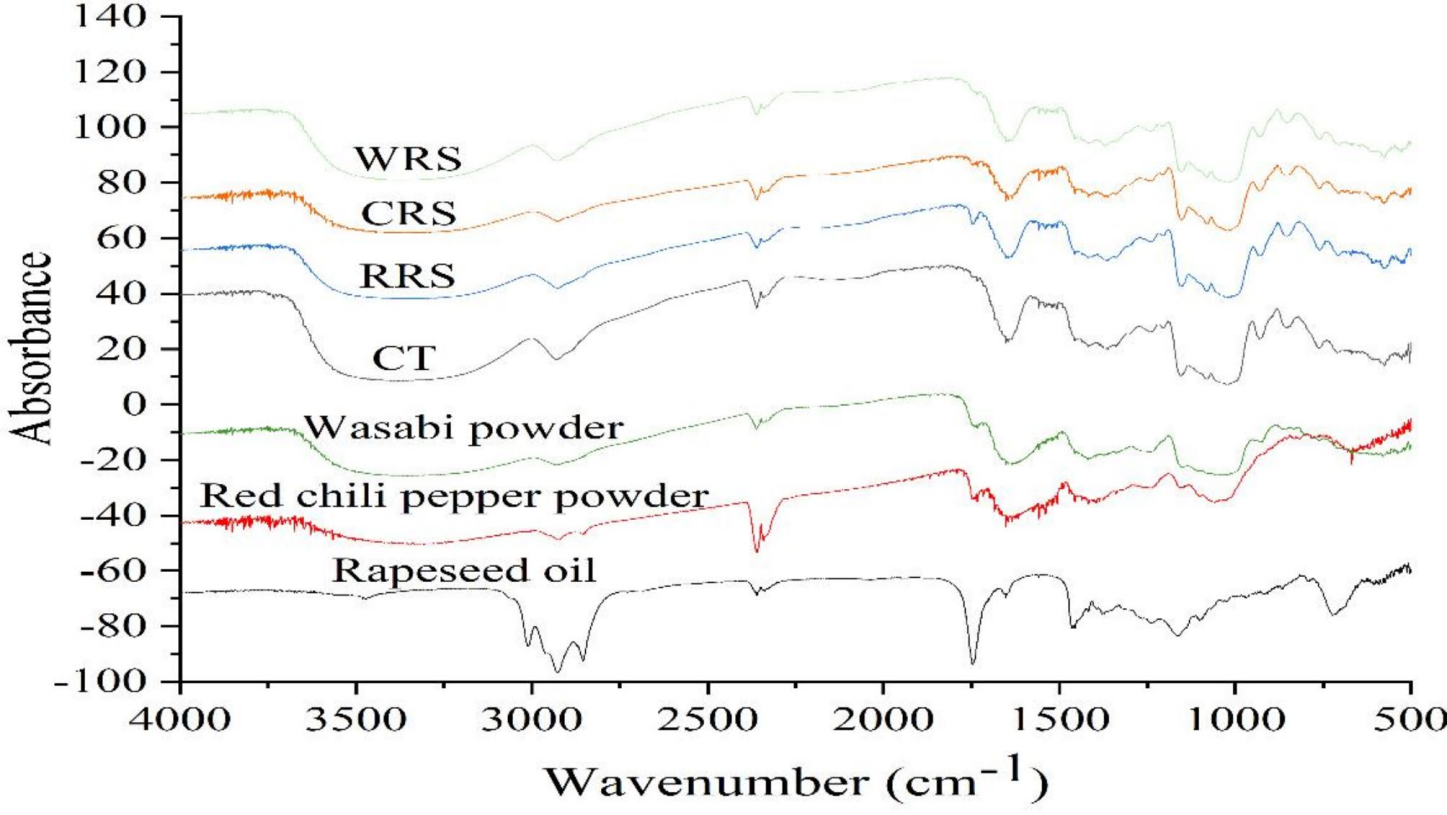
Fourier transform infrared spectroscopy spectra of cooked rice grains with different supplements. *CT* normal cooked rice (control), *RRS* cooked rice added with rapeseed oil, *CRS* cooked rice added with g dried chili pepper powder, *WRS* cooked rice added with dried wasabi powder.

FT-IR spectra of cooked rice, complexes as well as supplement alone are presented in Fig. 2. Different FTIR spectra was observed among different supplements. A high intensity peak around 3016 cm^−1^ was observed in rapeseed oil, which might relate to the unsubstituted polynuclear aromatic C–H stretching^42^. Similar results were reported by previous work^43^, noting that the spectral range from 3050 to 3000 cm^−1^ was related to the number of rings in aromatic compounds, whereas an opposite trend was detected between the number of rings and wavenumber. Characteristic transmittance peaks were identified around 2860 and 2964 cm^−1^, ascribed to asymmetric and symmetric stretching vibrations of CH_2_ and CH_3_ groups in fatty acids, and a peak at 1752 cm^−1^ due to the C = O in saturated esters^44^, as observed in rapeseed oil. In addition, the peaks around 2860 and 1752 cm^−1^ were observed in chili pepper powder and wasabi powder, which might correspond to the lipids retained within the seed. The peaks in the range of 1580–1720 cm^−1^ and 1480–1580 cm^−1^ were assigned as typical protein bands of amide I and amide II, respectively^45^. In the present study, these characteristic peaks were basically the same as those of complexes cooked, which indicated that covalent interactions did not form during cooking. The similar spectrum was reported when characterizing the interaction between corn starch and whey protein isolate (WPI)^46^. They observed strong bands around 1540 cm⁻¹ and 1655 cm^−1^ in WPI and all complex samples. Notably, a significant shift to lower wavenumber in the range of 3700 –3000 cm^−1^ was detected in the complexes of CRS and WRS, reflecting an increased hydrogen bond density and strength. Previous study^45^ reported a similar shift in spectral bounds of pea protein and chestnut starch complexes, from 3313 cm⁻¹ to 3274 cm⁻¹, following cooking treatment. This shift was attributed to the lower bond force constant of hydrogen bonds involving combined hydroxyl groups compared to free hydroxyl groups^11,47^. The stretching vibrations for primary and secondary alcoholic groups were shown in the range of 3100–3500 cm^−1^ as well as the skeletal mode vibration in α-(1–4) glycosidic linkage of the peak around 937 cm^−1^, which were common in all complexes and control group. Meanwhile, rice starch also showed IR patterns featuring characteristic transmittance peaks around 990 cm^−1^, corresponding to the C-O stretching vibrations of C-O-C glycosidic linkages in the polysaccharide. Peaks at 1640–1650 cm^−1^ were attributed to the O-H bonds involved in scissor bending vibration during starch hydration. In addition, two absorption peaks at 2856 and 1748 cm^−1^ were observed in all complexes as well as chili pepper powder and wasabi powder, which were ascribed to the asymmetric and symmetric stretching vibrations of –CH_3_ and –CH_2_ in the fatty acids and vibration of carbonyl. New signals appeared at 1683 and 1435 cm^−1^ in all complexes samples, which could be associated with a possible interaction between starch and polyphenols. Similar new signals at 1685 and 1447 cm^−1^ of gallic acid-rice starch complexes were observed by previous study^21^, while no such peaks were observed in samples of gallic acid or rice starch individually. Compared to the corresponding mixture, the FT-IR spectrum at 1654, 1558, 1547, 1512, 1460, 1385, 1240, and 1160 cm^−1^ had shifted and diminished, which implied that the interaction between the nutritional compounds and rice starch could prompt the shifting and disappearance of spectrum peaks. In summary, the variations among the FT-IR spectrum indicated that there might be differences among the main interactions between different supplements and rice starch.

### Morphology and tissue structure

**Fig. 3:**
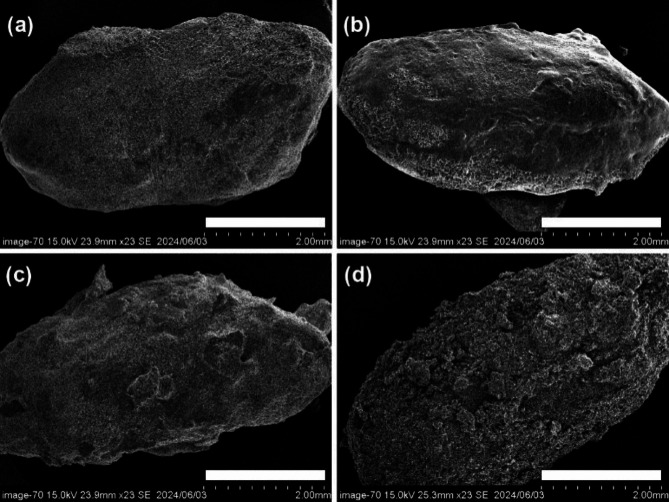
Morphological attributes of intact rice grains added with different supplements. (**a**) Normal cooked rice (CT); (**b**) cooked rice added with rapeseed oil (RRS); (**c**) cooked rice added with dried chili pepper powder (CRS); (**d**) cooked rice added with dried wasabi powder (WRS), scale bars indicate 2 mm.

**Fig. 4:**
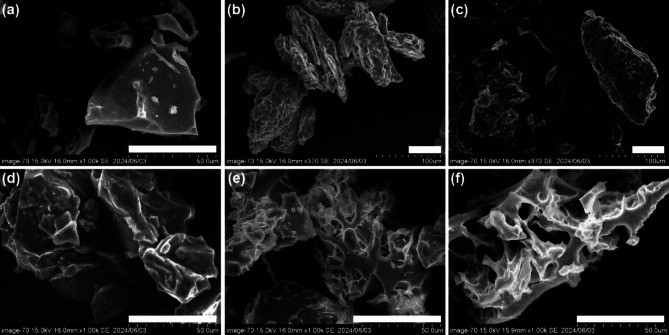
Morphological attributes of rice starch granules and supplements. (**a**) Normal cooked rice (CT); (**b**) dried chili pepper powder; (**c**) dried wasabi powder; (**d**) cooked rice added with rapeseed oil (RRS); (**e**) cooked rice added with dried chili pepper powder (CRS); (**f**) cooked rice added with dried wasabi powder (WRS), scale bars indicate 50 μm.

Scanning electron microscope (SEM) was used to investigate the effects of different supplements on the morphology of cooked rice (Figs. 3 and 4). Compared to CT, the cooked rice added with different supplements showed evident morphological differences. The CT samples exhibited relatively smooth spherical shape, alongside dense pore-like structures and cracks on the surface (Fig. 3a). The SEM images of rice supplemented with rapeseed oil revealed a transparent granular structure with a noticeable oil-enriched surface phase. Additionally, large protrusions and clusters were observed (Fig. 3b). The results indicated that the leached starch during the cooking process might interact with starch by the lipid crosslinking and agglomeration effect^48^. Previous studies reported that V-type resistant starch formed by crosslinking bonds between starch and lipids could retard starch digestion due to its ordered structure^29,49^. Moreover, characteristic agglomeration on CRS (Fig. 3c) and WRS (Fig. 3d) was also observed, which might be attributed to the noncovalent interactions with polyphenol and hydrogen bonding with protein among starch components^11,21^. Previous work reported reduced starch digestibility and an ordered structure where whey protein aligned in parallel with corn starch through hydrogen bonds^46^. This alignment promoted a more extensive hydrogen bond network and acted as a physical barrier against enzyme attack. These findings are in line with the FTIR and digestion analysis. Changes in the morphological micro-structure of granules were further determined (Fig. 4). Chili pepper powder showed stacked layers (Fig. 4b), while wasabi appeared conical with crumbly flakes (Fig. 4c). In contrast to the polygonal irregular shape of CT (Fig. 4a), the granular structure of RRS (Fig. 4d) showed aggregated and smooth structure due to the lipid cross-linking effect and oil-enriched surface^3^. A more aggregated cavity structure was exhibited in CRS (Fig. 4e) than RRS, which might due to the hydrogen bonds or van der Waals forces between starch and polyphenol and protein^50^. It is notable that WRS (Fig. 4f) showed distinct internal structures characterized by a looser, porous gel matrix with irregularly thick walls compared to the other complexes. This looser structure leads to easier enzyme access^51^, which was consistent with our starch hydrolysis results. Similar irregular pore-like gel matrix morphology was observed in rice starch-protein (casein, whey protein isolate, and soy protein isolate) and polyphenols (ferulic acid, gallic acid, and quercetin) complexes^11,21^, while the size of the grids varied depending on the type and of supplement polyphenol used. It suggested that nutritional compounds (e.g., polyphenol and protein type) probably dominated granular structure behavior in the mixture.

### Principal component analysis

**Fig. 5:**
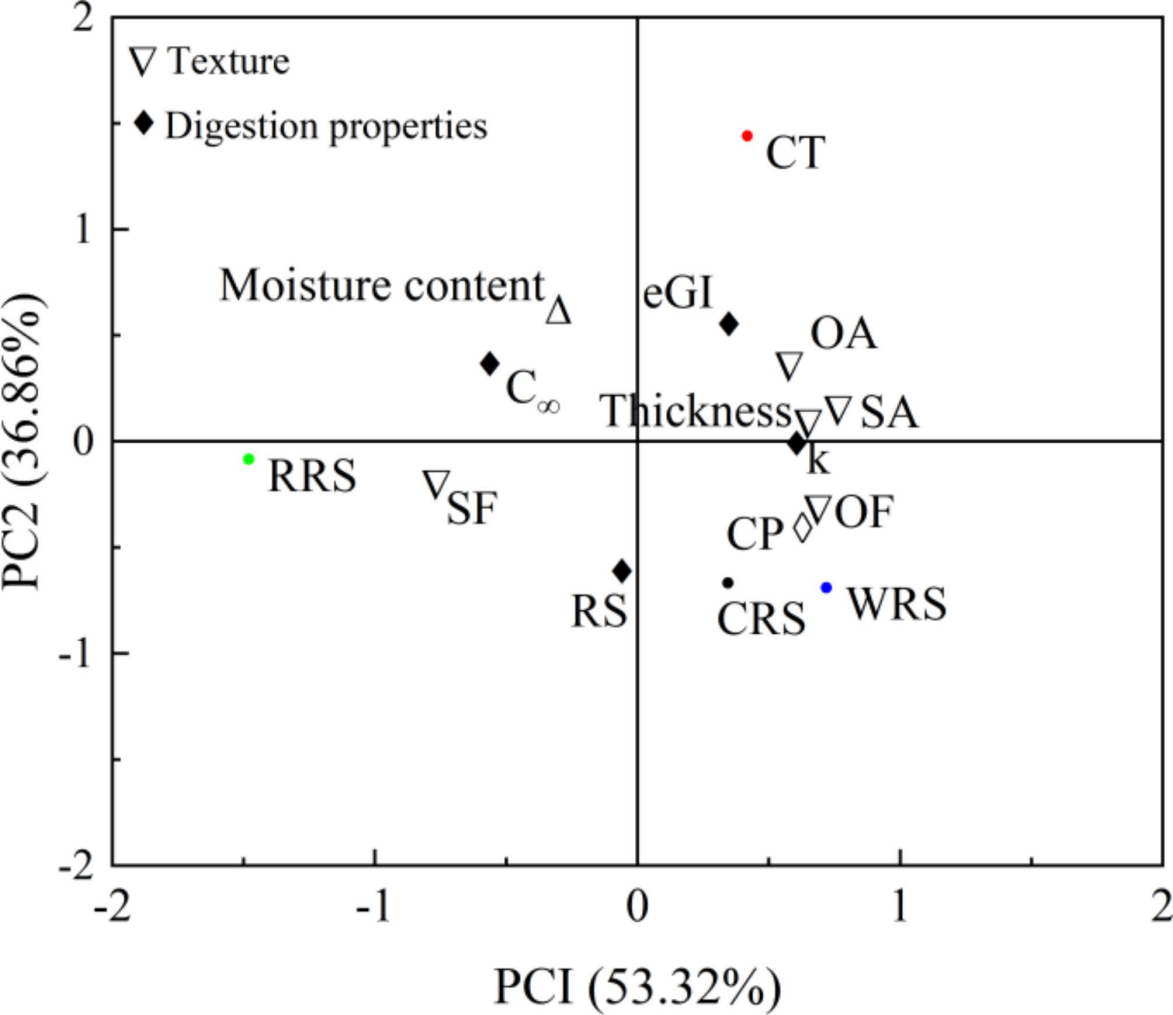
Loading plot of Principal component analysis (PCA) for the first two principal components of cooked rice samples with varied supplements. *CT* normal cooked rice (control), *RRS* cooked rice added with rapeseed oil, *CRS* cooked rice added with dried chili pepper powder, *WRS* cooked rice added with dried wasabi powder, *SF* surface firmness, *OF* overall firmness, *SA* surface adhesiveness, *OA* overall adhesiveness, *CP* crude protein content, *RS* resistant starch content, *eGI* estimated glycemic index.

To further explore a correlation among various supplements and physicochemical properties of cooked rice, PCA was established to obtain linear combinations of texture properties, moisture content, crude protein content, and in vitro digestibility properties. As shown in the loading plot (Fig. 5), the total data variance (with PC1 contributing 53.32% and PC2 contributing 36.86%) of 90.18% was elucidated. The projection of the CRS and WRS samples was clustered together in quadrants 4, while the CT and RRS samples were clustered in quadrants 1 and 3, respectively, indicating a clear difference among the samples based on various supplements. The samples added with supplements revealed the highest values of RS, SF, and OF, while negatively correlated with eGI and $\:{\text{C}}_{{\infty\:}}$, indicating that supplements might promote compact molecular structure, thus contributing to prevent enzyme approaches and interaction. Furthermore, the CRS and WRS had the highest values of CP content and OF, indicating that protein might play a more important role in constructing the order structure in these samples. These results confirmed the previous analysis.

## Conclusions

Molecular level investigations and starch hydrolysis kinetic parameters showed that the presence of supplements contributed to the clumping and crosslinking of starch, thus affecting structure and digestive characteristics. All samples added with supplements had increased SF and decreased thickness, which might be attributed to the swelling and gelatinization influenced by the lipid layer and interactions with guest molecules (e.g., fatty acids, polyphenols, proteins, etc.) as well as the migration of water. Starch hydrolysis kinetic parameters of $\:{\text{C}}_{{\infty\:}}$, k and eGI of the samples were influenced by supplements significantly. The addition of supplements increased the levels of resistant starch (RS) in all samples. FT-IR and SEM results revealed that interactions were primarily driven by noncovalent bonds, where the guest chemistry component could influence rice starch to form short-range ordered structure in crystalline regions (e.g., V-amylose, starch-protein/polyphenol complexes). These results were consistent with principal component analysis (PCA) analysis. This study extends a better understanding of starch-supplements interactions and their effects on the multi-scale structures, which could facilitate the applications of varied supplements to develop novel starchy functional foods. Since this study has mainly focused on the effect of supplement types on the attributes of cooked rice, further work should test whether the concentration of supplements and their composition are related to the low digestibility.

## Methods

### Materials and chemical reagents

White rice (*Oryza sativa* L. cv. Koshihikari, Japan), rapeseed oil, dried chili pepper, and wasabi were purchased from local supermarkets (Matsudo, Chiba, Japan). The glucose assay kit was purchased from Megazyme International Ireland Ltd. (Wicklow, Ireland). Pancreatin (hog pancreas, activity 8 × USP), pepsin (porcine gastric mucosal, ≥ 250 mg^−1^ solid), and invertase (grade VII, ≥ 300 mg^−1^ solid) were purchased from Sigma-Aldrich Co., Ltd. (St. Louis). All other analytical grade reagents were purchased from Dojin Chemical Laboratory Co. Ltd. (Tokyo, Japan).

### Sample preparation

All chosen supplements were based on the common eating habits in Asia. The detailed preparation method was according to previous research^19,24^. Rice grains (400 g) were cooked with 600 mL deionized water using a rice cooker (TK-RC12, Eupa, Tokyo, Japan) for 25 min. Afterwards, the cooked grains were separated into 100 g each, and each supplement was mixed with the cooked rice at a concentration of 10% dry basis (≈ 4–5% wet basis). The resulting samples were designated as follows: cooked rice with added rapeseed oil (RRS), cooked rice with added dried chili pepper powder (CRS), and cooked rice with added dried wasabi powder (WRS). Rice cooked without any added additives was designated as the control group (CT). All samples were placed in petri dishes and covered with plastic wrap to allow moisture equilibration in an incubator set at 35 °C for 25 min. The in vitro digestibility was determined instantly after equilibrium. Meanwhile, the measurement of moisture and texture was completed within 20 min. The remaining samples were stored overnight in a -80 °C refrigerator and then freeze-dried. The obtained samples were grinded into powder and passed through a 200-sieve mesh. The rice flour was stored in a sealed bag at 4 °C for further analysis.

### Moisture content

Approximately 5.0 ± 0.2 g of cooked grains and 3.0 ± 0.2 g of rice powder were dried at 135 °C for 36 h in an air oven (Oven 8150, Labserv, Longford, Ireland) according to Tamura et al.^30^, and the moisture content (% w.b.) was calculated as the weight loss (%) before and after drying using the following formula:

$$\:\text{Moisture content}\:\left(\%\right)=({\text{W}}_{2}-{\text{W}}_{1})/{\text{W}}_{2}\times\:100$$

where W_2_ and W_1_ are the weight of the sample before and after drying, respectively.

### Crude protein (CP) content

The nitrogen content of the powdered sample (0.15 g) was determined using a CN Coder (MT-700, Yanaco, Kyoto, Japan). A nitrogen–protein conversion factor (5.95) was used to calculate the crude protein content from nitrogen content results. Hippuric acid (200-37032) was purchased from Kishida Chemical (Osaka, Japan) and was used as the standard nitrogen material^19^.

### Texture analysis

A creep meter (RE2-3305 S, Yamaden Co. Ltd., Tokyo, Japan) equipped with a 20 kg·m/s^2^ (N) load cell was used to determine the texture characteristics of cooked samples. The compression test mode was defined as the surface (25% of initial thickness) and overall (90% of initial thickness) when measuring firmness and adhesiveness. A single cooked grain was placed on a cylindrical baseplate and compressed twice using a planar plunger (Ø56 mm) at a speed of 1 mm/s with the contact point between the plunger and grain set as 0.02 N trigger force. Two positive curves (surface firmness and overall firmness) and two negative curves (surface and overall adhesiveness) for the texture profile were obtained^24^. The thickness of the sample was recorded as the distance between the plunger and baseplate. Approximately 15 replicates were completed within 20 min to maintain the moisture and textural properties during testing.

### Simulated in vitro gastro-small intestinal digestion

The total starch (TS) contents of the powdered rice flour were measured according to the total starch assay kit (K-TSTA 07/11, Megazyme International, Wicklow, Ireland) based on AOAC Method 996.11, AACC Method 76–13.01 and previous study^24,53^. Simulated gastric fluid (SGF) and simulated intestinal fluid (SIF) were prepared according to a previously reported method^52^. A magnetic stirrer (color squid white, IKA, Staufen, Germany) was used to continuously agitate the sample (200 rpm) in the reactor. Cooked rice grains were placed into a net bag and then placed in a jacketed glass reactor at 37 °C. Then, 17 g of 4% total starch equivalent cooked rice grains were subjected to a two-stage simulated gastro-intestinal in vitro digestion model^53^. The experimental conditions have been described in detail by Dartois et al.^52^ and Tamura et al.^53^. The pH of gastric digestion was adjusted to 1.20 ± 0.02 with 6 M HCl at the initial gastric phase (G0) and then with 1 M HCl at subsequent stages (G15, G30). The digestive solution (0.5 mL) was collected at 5, 15, and 30 min. After 30 min of gastric digestion, 23 mL of SIF was added to simulate intestinal digestion, and the pH was maintained at 6.80 ± 0.02 with 3 N and 1 N NaOH. The digestive solution (0.5 mL) of the small intestinal digestion phase was collected at 5, 15, 20, 30, 60, 90, 120, 180, 240, 300, 360, and 450 min. All collected digestive solutions were placed in a tube, and the digestive enzyme activity was terminated by thoroughly mixing the samples with 3 mL of 95% ethanol. The collected samples were centrifuged (1800×g, 10 min) and incubated with amyloglucosidase and invertase at 37 °C for 10 min. The glucose concentration was measured using a D-glucose assay kit (GOPOD, Megazyme International Ireland). All calculations were based on the dry basis of starch. The results of starch hydrolysis were calculated as percentage (%) following the equation:

$$\:{\% \text{S}}_{\text{H}}={\text{S}}_{\text{h}}/{\text{S}}_{\text{i}}=0.9\times\:{\text{G}}_{\text{p}}/{\text{S}}_{\text{i}}$$

where %S_H_ is the percentage of starch hydrolysis, S_h_, S_i_ and G_p_ are the amount of hydrolyzed starch, initial TS, and the glucose produced, respectively. A conversion factor of 0.9 was used, which was calculated from the molecular weight of the starch monomer against that of glucose (162/180 = 0.9)^54^.

### Resistant starch content

The digestion resistant fraction was determined according to the AOAC Method, using the RS assay kit (K-RSTAR; Megazyme International Ireland). About 100 mg of powder was weighed into a screw cap tube and incubated with pancreatic α-amylase (10 mg/mL) and AMG (3 U/mL) at 37 °C for 16 h. This process aimed to hydrolyze and solubilize non-resistant starch components. Four milliliters of ethanol (99% v/v) was added to terminate the reaction, then the recovered resistant starch was centrifuged and washed with 2 M KOH. Afterwards, 8 mL of 1.2 M sodium acetate buffer (pH 3.8) was added to neutralize the high alkalinity of the obtained solution, and AMG was used to quantitatively hydrolyze the resistant starch fraction. The glucose was determined by glucose oxidase/peroxidase (GOPOD) reagent. The contents of RS were calculated as suggested by the manufacturer’s protocol.

### Dynamic analysis of in vitro enzymatic hydrolysis

A non-linear first-order equation model was used to calculate the kinetics of starch hydrolysis as described below^54^:

$$\:\text{C}={\text{C}}_{{\infty\:}}\left(1-{\text{e}\text{x}\text{p}}^{-\text{k}\text{t}}\right)$$

where C and $\:{\text{C}}_{{\infty\:}}$ represent the hydrolysis percentage at digestion time t and the equilibrium percentage of starch during the simulated intestinal digestion phase, respectively, and k is the kinetic constant.

The fitting quality of the first-order equation model was evaluated using the determination coefficient (R^2^).

$$\:{R}^{2}=1-\frac{{{\sum\:}_{\text{i}=1}^{N}({C}_{exp,\:\text{i}}-{C}_{pre,\:\text{i}})}^{2}}{{{\sum\:}_{\text{i}=1}^{N}({C}_{exp,\:\text{i}}-{C}_{ave})}^{2}}$$

where C_exp, i_ and C_pre, i_ represent the experimental and predicted percentages of hydrolyzed starch from the “i”-th experiment, respectively. Cave is the average experimental percentage of hydrolyzed starch, and N represents the number of observations.

The hydrolysis index (HI) was defined as the area under the hydrolysis curve of cooked grain samples divided by that of white bread. The estimated glycemic index (eGI) was calculated using the formula from^54^:

$$\:\text{e}\text{G}\text{I}=39.71+0.549\text{H}\text{I}$$

### Fourier transform infrared spectroscopy (FTIR)

To characterize the structural changes induced by supplements in rice starch, the FTIR spectra of freeze-dried powder samples and supplements were recorded using a Fourier transform spectrophotometer (FT/IR-4200ST + IRT-5000, Jasco Corporation, Japan).

The spectra were scanned in the range of 4000–400 cm^−1^. Sixty-four accumulation scans were obtained at a resolution of 2 cm^−1^^9^. A resolution enhancement factor of 1.5 and half-band width of 15 cm^−1^ were employed.

### Morphology and tissue structure

The microstructural changes of grain samples under external and internal conditions were observed using Scanning Electron Microscopy (SEM) (SU1510; Hitachi High-Tech, Tokyo, Japan). The freeze-dried samples were mounted on double-sided adhesive tape and observed in high vacuum mode at an accelerating voltage of 5 kV. The obtained images were analyzed by graphic software (Photoshop, Adobe, San Jose, CA, USA).

### Statistical analysis

Statistical data and differences based on at least triplicate measurements were presented as means ± standard deviations. Analysis was conducted using the t-test (IBM SPSS Statistics, version 25.0; IBM Corp., Armonk, NY, USA), with a priori significance level set at *p* < 0.05 considered statistically significant. Analysis of variance (ANOVA) was also conducted. The principal component analysis (PCA) was performed using Origin 2018 (Origin Lab, California, USA).

## Data Availability

Data sets generated during the current study are available from the corresponding author on reasonable request.
